# Association of Atrial Fibrillation Recurrence with Right Coronary Atherosclerosis and Increased Left Arterial Epicardial Fat Following Catheter Ablation—Results of a Multimodality Study

**DOI:** 10.3390/life13091891

**Published:** 2023-09-10

**Authors:** Lehel László Bordi, Theodora Benedek, István Kovács, Diana Opincariu, Emese Márton, Zsolt Parajkó, Renáta Gerculy, Imre Benedek

**Affiliations:** 1 Clinic of Cardiology, Mures, County Emergency Clinical Hospital, 540142 Targu Mures, Romania; 2 Center of Advanced Research in Multimodality Cardiac Imaging, CardioMed Medical Center, 540124 Targu Mures, Romania; 3 Doctoral School of Medicine and Pharmacy, University of Medicine, Pharmacy, Science and Technology “George Emil Palade” of Targu Mures, 540139 Targu Mures, Romania; 4 Faculty of Medicine and Pharmacy, University of Medicine, Pharmacy, Science and Technology “George Emil Palade” of Targu Mures, 540139 Targu Mures, Romania

**Keywords:** atrial fibrillation, predictors for AF recurrence, left atrium diameter, calcium score, left atrial volume index

## Abstract

Background: Identification of predictors for atrial fibrillation (AF) recurrence after pulmonary vein isolation (PVI) can lead to better long-term results. Our aim was to investigate the association between novel CT imaging markers reflecting the severity of coronary atherosclerosis and the risk of recurrence following PVI. Methods: This study included 80 patients with paroxysmal/persistent AF who underwent PVI. The patients were divided into two groups: Group 1–23 patients with recurrence and Group 2–57 patients without recurrence. Results: Patients with recurrence presented with a more enlarged left atrial diameter and reduced left ventricle EF, as assessed by echocardiography. Elevated calcium scores and right coronary artery (RCA) stenosis were correlated with a higher risk of AF recurrence (25.38 ± 4.1% vs. 9.76 ± 2.32%, *p* = 0.001). Patients with AF recurrence presented a higher left atrial volume index (LAVI) (61.38 ± 11.12 mm^3^/m^2^ vs. 46.34 ± 12.27 mm^3^/m^2^, *p* < 0.0001). The bi-atrial volume index (BAVI) was similarly higher in the AF recurrence group (98.23 ± 14.44 mm^3^/m^2^ vs. 76.48 ± 17.61 mm^3^/m^2^, *p* < 0.0001). Increased EAT volumes located around the LA (EAT-LA) were correlated with recurrence (25.55 ± 6.37 vs. 15.54 ± 8.44, *p* < 0.0001). Conclusions: RCA stenosis, together with atrial volumes and EAT-AS evaluated by CCTA, is associated with the risk of AF recurrence following PVI.

## 1. Introduction

Atrial fibrillation (AF) has a high prevalence and incidence globally, making it the most common type of sustained cardiac arrhythmia [1], causing a remarkable burden for the healthcare system [2]. Until it was established that the pulmonary veins are one of the main triggers of AF and before the introduction of catheter ablation, AF was not considered a curable disease [3].

Rhythm control strategies, with pulmonary vein isolation (PVI) by radiofrequency ablation (RFA) or cryoballoon ablation (CBA), have become widely accepted and effective treatment options for patients with symptomatic AF and in cases of refractory to antiarrhythmic treatment [4]. 

The EAST-AF trial evaluated the effectiveness of early, systematic rhythm control therapy, including AF ablation, in patients with symptomatic and asymptomatic AF. As part of an early, systematic rhythm control approach, ablation therapy can provide comparable clinical benefits for both symptomatic and asymptomatic patients with recently diagnosed AF [5]. The same benefits were demonstrated in the EARTLY-AF trial by Jason G. et al., which provided evidence that CBA is superior to antiarrhythmic drugs in reducing the recurrence of AF in subjects with symptomatic and paroxysmal AF [6].

Ablation therapy is an invasive therapeutic procedure that can be associated with high costs and possible periprocedural complications. Proper patient selection plays an important role in reducing the risk of arrhythmia recurrence [7]. The success rates after pulmonary vein isolation range from 50% to 70%, depending on the type of AF and ablation technique. Patients with paroxysmal AF tend to have higher rates of success (70%), while those with persistent AF have success rates closer to 50% [8,9]. 

Several studies have aimed to identify and manage predictors and risk factors for AF recurrence following PVI. The main objectives were to improve patient selection and establish better treatment strategies, reducing the risks and costs of inefficient therapies [10]. 

The coronary artery calcium score (CS), evaluated by CCTA, is a very accessible, non-invasive imaging method. It is considered a direct marker of coronary atherosclerotic burden and a powerful predictor of major adverse cardiovascular events (MACE) regardless of general risk factors, sex, and age [11]. Several studies have demonstrated a significant association between CS and AF appearance. Elevated coronary artery CS has been described to correlate with a higher risk of AF appearance, particularly if above 100 UI Agatson [12]. Coronary CS is increasingly used as a risk predictor for acute cardiac events, but there are limited data available on its impact on AF recurrence following ablation.

It has been proven that AF leads to anatomical and functional remodeling of the atrium. These changes have an important role in the induction and progression of the arrhythmia. The extent of left atrial (LA) enlargement indicates the anatomic remodeling of the LA. Thus, it may predict the risk of arrhythmia recurrence, among other imaging parameters [13].

Some studies have reported that M-mode LA diameter is associated with AF recurrence after ablation therapy [14]. Nevertheless, these results have not been constant across other studies [15]. LA dimension, determined by echocardiography, is usually used in clinical practice, but it has inadequate accuracy for measuring LA diameter due to the irregular geometry of the atrial endocardium and the technical features of 2D echocardiography. 

Imaging the LA anatomy, pulmonary vein (PV) variations, and related intrathoracic structures before ablation is useful in guiding the procedure [16]. CCTA allows 3D visualization of cardiac anatomy, including the LA, the number and variations in pulmonary veins, as well as the LA appendage with possible thrombi in it. Another main role of CCTA before radiofrequency ablation is that imaging data are integrated with advanced electro-anatomical mapping systems (Ensite NavX) to guide the operator during the ablation procedure [17].

In addition, CCTA-derived parameters have been shown to predict AF recurrence after ablation as well as after cardioversion. LA size assessed by CCTA is a well-known risk factor for AF recurrence after pulmonary vein isolation [18]. Augustin et al. demonstrated in their study that patients with AF recurrence post-AF ablation had significantly higher left atrial volume (LAV) and left atrial volume index (LAVI) compared to patients without recurrence [19]. Other imaging parameters include right atrial volume, left atrial appendage morphology, pulmonary vein anatomy, and other structural cardiac disorders [20]. 

The aim of the current study was to investigate the role of echocardiographic and CCTA imaging parameters acquired during the preprocedural workup of patients undergoing pulmonary vein isolation for paroxysmal and persistent AF in correlation with the risk of arrhythmia recurrence over a one-year follow-up period.

## 2. Materials and Methods

### 2.1. Study Population

In this prospective, observational, non-randomized, single-center study, we investigated the role of CCTA and echocardiography-derived parameters in correlation with the AF recurrence risk following PVI. This study included 80 consecutive patients who met the inclusion criteria and underwent successful PVI procedures, either by CRA or by RFA, in the Cardiology Department of the Emergency Clinical County Hospital of Târgu Mureș, Romania, between 2017 and 2021, with a follow-up period of 12 months. The inclusion criteria for the study comprised patients diagnosed with non-valvular paroxysmal or persistent atrial fibrillation who were able to provide informed consent and were at least 18 years of age. Patients with permanent or long-standing persistent AF, redo ablation procedures, patients in whom arrhythmia was supposably triggered by hyperthyroidism, those who had acute coronary syndromes in the last 30 days, subjects with diagnosed malignancy, autoimmune diseases, allergies to iodine contrast, or patients during pregnancy or lactation were excluded from the study. 

**Figure 1:**
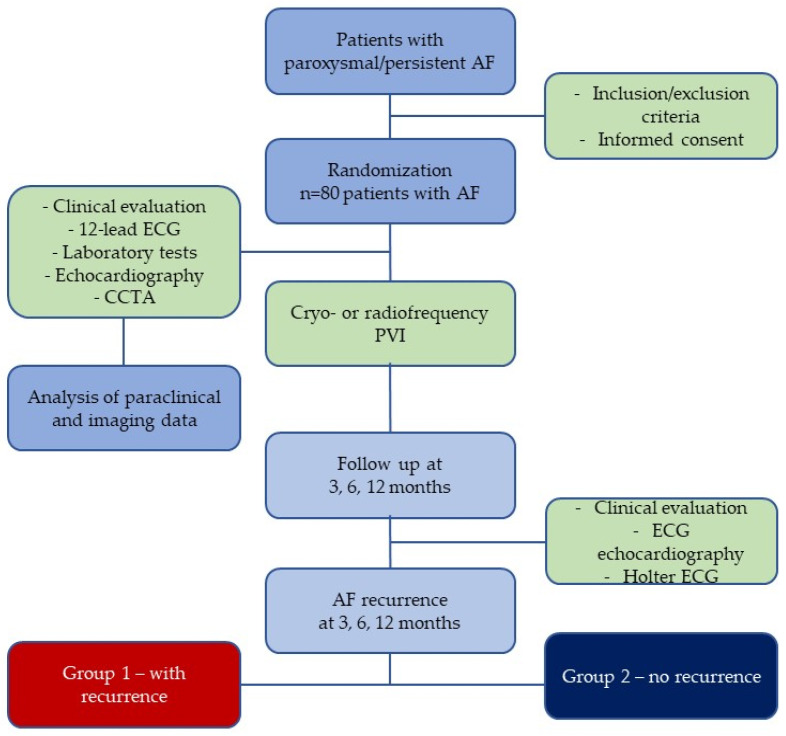
Flowchart diagram of the study protocol.

At baseline, data regarding demographic and anthropometric measurements (gender, age, weight, height, body surface area, and body mass index), medical history, and cardiovascular risk factors were recorded. General laboratory parameters, a twelve-lead ECG, echocardiography, and CCTA were performed for all subjects before the procedure. All patients underwent primary PVI, with 40 patients undergoing CRA and 40 patients undergoing RFA. Redo procedures were not included in the current analysis. The patients were divided into two distinct groups according to AF recurrence during the 1-year overall follow-up period. Group 1 had 23 patients who had AF recurrence, and Group 2 had 57 patients who had no recurrence during the 12-month follow-up period (Figure 1). 

### 2.2. Study Procedures

#### 2.2.1. Transthoracic Echocardiography

An echocardiographic examination was conducted after the procedure and at 1, 3, 6, and 12 months post-ablation using a Vivid E9 echocardiography device (General Electric Vingmed Ultrasound, Horten, Norway). Echocardiographic parameters were obtained in the standard two-dimensional parasternal long and short axis and apical two- and four-chamber views. The LV end-diastolic and end-systolic diameters, LV septal and posterior wall thickness, and LA antero-posterior diameter were measured. The LV ejection fraction was established utilizing Simpson’s biplane method in images acquired in the apical four- and two-chamber views. 

#### 2.2.2. CCTA Analysis

**Figure 2:**
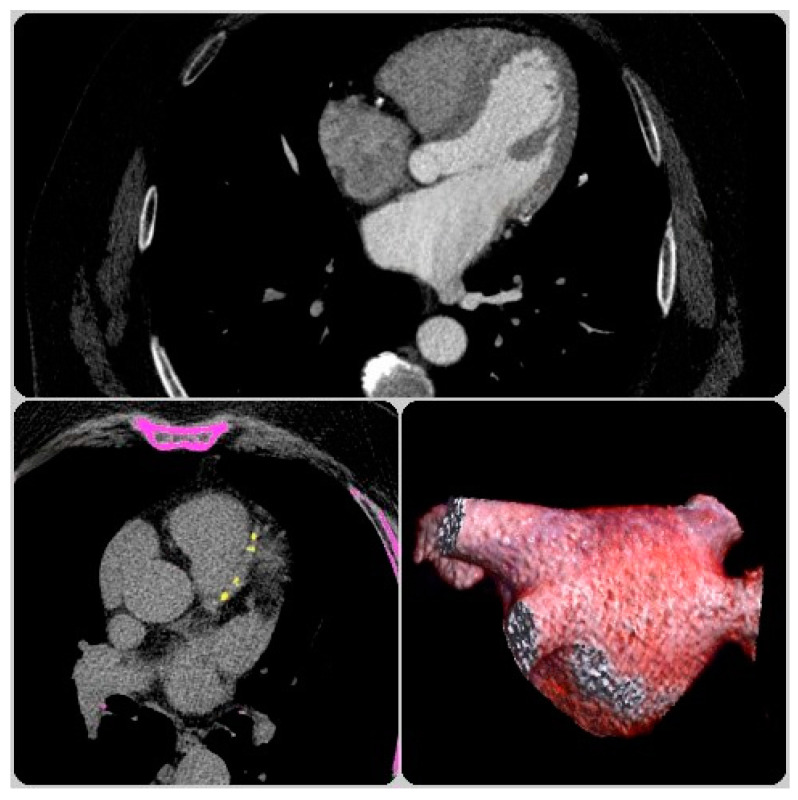
Three-dimensional reconstruction of the left atrium and CS determination by CCTA.

The CCTA images were acquired with 64-slice (Somatom Sensation 64-slice CT) and 128-slice CT (Somatom Definition 128-slice CT, Siemens Healthcare, Germany). A native scan was used for the evaluation of coronary CS, followed by a retrospective ECG-gated contrast-enhanced acquisition protocol for the evaluation of atrial anatomy and coronary artery multiplanar reconstruction. Image post-processing was performed offline using Syngo via Frontier software VB10B (Siemens Healthineers, Erlangen, Germany). Coronary artery calcification was obtained using a standard native scanning protocol based on Agatston scoring. After the exclusion of left atrial appendage thrombosis, LA and RA areas were manually traced on axial and sagittal planes during the end-systolic phase at 60, 70, or 80% RR intervals. For each CCTA data set, the structural characteristics of the left atrium and pulmonary veins were assessed by manually tracing the endocardial border from the LA roof to the level of the mitral annulus, with the exclusion of the left atrial appendage at the base and the PV at the ostial level. For right atrial dimensions, the RA area was traced from the roof to the tricuspid annulus, with the exclusion of the superior and inferior caval veins and coronary sinus at their respective ostia. The left and right atrium volumes were automatically calculated based on 3D reconstruction and reported with Syngo via Frontier software for atrial volume calculation (Figure 2). Dividing the atrial volumes by the body surface area, the indexed atrial volume was obtained. The body surface area was determined using the Dubois formula, which took into account the patient’s height and weight. The measured LA and RA volumes were added and indexed to the total body surface area for the calculation of the bi-atrial indexed volume. 

The epicardial adipose tissue (EAT) was measured within a range of −50 to −150 HU. The volume of EAT around the LA (EAT-LA) and bi-atrial (EAT-BA) was quantified using 2 mm axial slices at the end of the atrial diastole. The volumes were obtained by manually contouring in each section around the posterior pericardium of the LA, from the mitral valve annulus to the roof of the LA, and around the posterior pericardium of the RA, from the level of the inferior vena cava ostium to the roof of the RA. 

#### 2.2.3. Ablation Protocols

RFA was performed using the Seldinger technique through the right femoral venous approach. A diagnostic catheter was placed in the coronary sinus, while a transseptal sheath was inserted at RA level. Under the guidance of transesophageal echocardiography (ETE) and fluoroscopy, a transseptal puncture was performed, allowing the insertion of the ablation catheter and a flexible diagnostic duodecapolar catheter at the level of LA for electrical signal detection for mapping. A 3D electro-anatomical map was reconstructed using integrated images from CCTA in the EnSite NaVX system (St. Jude Medical, St. Paul, MN, USA). The isolation procedure was performed at the pulmonary vein ostium using the contact-force irrigated catheter, applying optimal pressures between 10 and 30 g during the isolation process. The ablation procedure was considered successful when complete electrical blockage was achieved between the atrial myocardium and the pulmonary veins.

In the case of CRA, the right femoral vein was also applied as an approach. Following the guided transseptal puncture using ETE and fluoroscopy, a special FlexCath Advance™ steerable sheath was used, which facilitated easier maneuvering of a decapolar diagnostic catheter and the cryoballoon to the level of the LA. Isolation of the pulmonary veins was performed consequently using cryoballone (Arctic Front Advance™, Medtronic), with each PV being treated for a duration of up to 240 s at an average temperature of −47 degrees Celsius. Successful isolation was defined as achieving complete electrical blockage between the LA and PV following the procedure. If reconnection was detected, CRA was repeated for an additional 180 s.

#### 2.2.4. Patient Follow-Up

Follow-up assessments were performed at 1, 3, 6, and 12 months post-procedure. Each follow-up evaluation was a physical visit with clinical, laboratory, and echocardiography examinations, followed by 48-h Holter monitoring of the patients. If patients reported symptoms suggestive of arrhythmia during the period between two follow-up visits, the Holter examination was repeated at a two-week interval. The specific symptoms considered suggestive of arrhythmia in our study included palpitations, irregular heartbeats, dizziness, fainting (syncope), and unexplained episodes of fatigue or shortness of breath. The definition of AF recurrence involved both clinical reliance on patient reporting of symptomatic recurrences and detection of AF episodes on 24-h Holter ECG monitoring.

### 2.3. Statistical Analyses

Post-hoc statistical data analysis was performed by using the MedCalc statistical software version 20.2.18 (MedCalc Software Ltd., Ostend, Belgium) for computing the ROC curve analysis. For uni- and multivariable analysis, GraphPad Prism 7 (GraphPad Software, San Diego, CA, USA) was employed. Continuous variables were expressed as mean ± standard deviation (SD) and median. Qualitative values were expressed as integer values and percentages. For the assessment of the Gaussian distribution, the D’Agostino–Pearson test was used. The statistical threshold for significance was set at *p* < 0.05.

### 2.4. Ethics

The research protocol received approval from the Ethics Committee for Scientific Research of the University of Medicine, Pharmacy, Sciences and Technology “George Emil Palade” of Targu Mures. Before being enrolled in the study, all subjects were evaluated for eligibility based on the inclusion and exclusion criteria and with informed consent provided. All study procedures were conducted in compliance with the principles outlined in the Declaration of Helsinki of 1975.

## 3. Results

Patients were divided into two groups according to the recurrence of AF occurring after the first 3 months, which is considered the blanking period. Among the 80 patients treated with catheter ablation, 19 (23.7%) presented recurrence at 6 months (9 treated with cryoablation and 10 with radiofrequency ablation) and 23 (28.7%) at 1 year (11 treated with cryoablation and 12 with radiofrequency ablation). All patients received anti-lipid treatment according to their risk profile after defining the target value for LDL cholesterol as stated in the guidelines [21]. This consisted of high-intensity statins (atorvastatin or rosuvastatin) in 36 cases or statin + ezetimib in 17 cases. There were no significant differences between the study groups regarding the use of statins. At the same time, all patients received beta-blockers or amiodarone for 6 months following ablation, as per standard local protocol.

The demographic data, cardiovascular risk assessment, type of AF, and medical history of the study population are summarized in Table 1. Patients with AF recurrence presented a significantly higher mean age than those free of AF during follow-up (60.35 ± 9.2 years in Group 1 and 55.4 ± 9.2 years in Group 2, *p* = 0.03). No statistically significant differences were observed between the two groups in terms of gender (*p* = 0.05), medical history of coronary artery disease (*p* = 0.4) or previous stroke (*p* = 0.9), or comorbidities such as hypertension (*p* = 0.2), diabetes (*p* = 0.9), obesity (*p* = 0.7), dyslipidemia (*p* = 0.3), and smoking (*p* = 0.6). Regarding the type of AF, arrhythmia recurrence was more common in patients with persistent AF but without significant statistical differences between the types (persistent 65.2% vs. paroxysmal 34.8%, *p* = 0.2). There was no significant difference between the two groups regarding the lipid-lowering (*p* = 0.09), antihypertensive (*p* = 0.7), antiarrhythmic (*p* = 0.4), and anticoagulant (*p* = 0.8) medical treatment of the patients.

Comparison of the echocardiographic parameters between the two study groups showed a higher LA diameter in the recurrence group (45.3 ± 4.29 mm vs. 41.25 ± 5.94 mm, *p* = 0.003), as well as a significantly lower ejection fraction (51.26 ± 4.58% vs. 53.14 ± 5.54, *p* = 0.03) and a higher estimated systolic pulmonary artery pressure (28.28 ± 7.7 mmHg vs. 24.53 ± 6.49 mmHg, *p* = 0.016). However, left ventricular end-diastolic and end-systolic diameters, interventricular septum and posterior wall thickness, right ventricle diameter, and the late end-early mitral diastolic velocity parameters were similar between the two study groups. Echocardiographic features are represented in Table 2.

**Figure 3:**
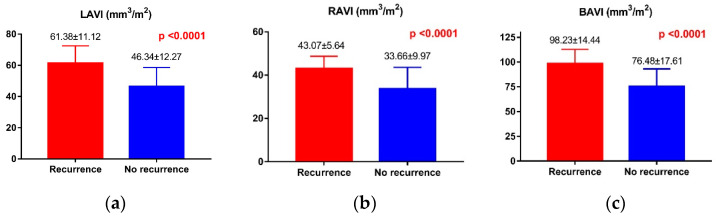
Atrial volumes and AF recurrence in the study groups. (**a**) Left atrial indexed volume and AF recurrence; (**b**) Right atrial indexed volume and AF recurrence; (**c**) Bi-atrial indexed volume and AF recurrence.

The CCTA parameters are summarized in Table 3. CCTA examination revealed that subjects who experienced AF recurrence had a statistically significant increase in left atrial volume (108.7 ± 21.25 mm^3^ vs. 83.79 ± 19.94 mm^3^, *p* = 0.0002) and higher indexed left atrial volume (61.38 ± 11.12 mm^3^/m^2^ vs. 46.34 ± 12.27 mm^3^/m^2^, *p* < 0.0001). In addition, the group with AF recurrence had significantly higher right atrial volume, higher indexed right atrial volume (43.07 ± 5.64 mm^3^/m^2^ vs. 33.66 ± 9.97 mm^3^/m^2^, *p* < 0.0001), and higher indexed bi-atrial volume (98.23 ± 14.44 mm^3^/m^2^ vs. 76.48 ± 17.61 mm^3^/m^2^, *p* < 0.0001). The correlation regarding the calculated indexed atrial volumes between the groups is illustrated in Figure 3.

The AF recurrence group had a higher total calcium score (136.8 ± 37.29 vs. 31.56 ± 7.75, *p* = 0.02). When measured across the three main coronary arteries, CCS was similar in the left anterior descending (group 1—44.86 ± 15 vs. group 2—15.5 ± 5.47, *p* = 0.09) but significantly higher in the left circumflex (group 1—20.97 ± 7.71 vs. group 2—2.18 ± 0.9, *p* = 0.03) and right coronary artery (group 1—73.97 ± 22.83 vs. group 2—8.88 ± 3.08, *p* = 0.005) in patients that were prone to arrhythmia recurrence after ablation (Table 3 and Figure 3).

**Figure 4:**
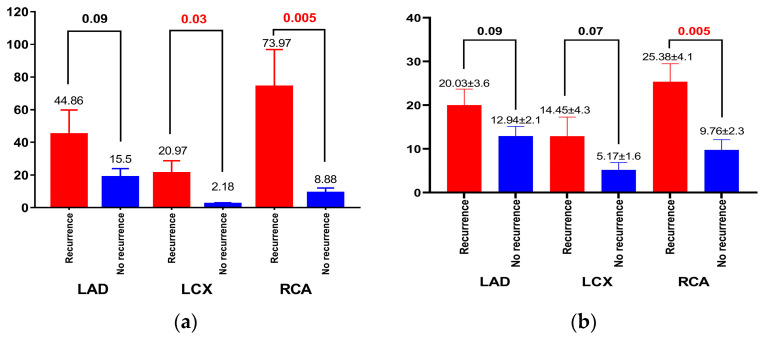
(**a**) Calcium score and AF recurrence for each coronary artery; (**b**) Stenosis for each coronary artery and AF recurrence.

Statistical results in the evaluation of coronary stenosis did not show significant differences between the two groups regarding stenosis at the level of the LAD (20.03 ± 3.66% vs. 12.94 ± 2.16%, *p* = 0.09) and LCX (12.91 ± 4.34% vs. 5.17 ± 1.69%, *p* = 0.07). However, we observe that patients with AF recurrence had significantly higher stenosis at the level of the RCA compared to patients without recurrence (25.38 ± 4.1% vs. 9.76 ± 2.32%, *p* = 0.005) (Table 3, Figure 4).

The prognostic value of atrial volumes assessed by CCTA was evaluated by performing an ROC curve analysis. Table 4 displays the cut-off values of atrial volumes for AF recurrence, with their associated area under the curve, specificity, and sensitivity.

**Figure 5:**
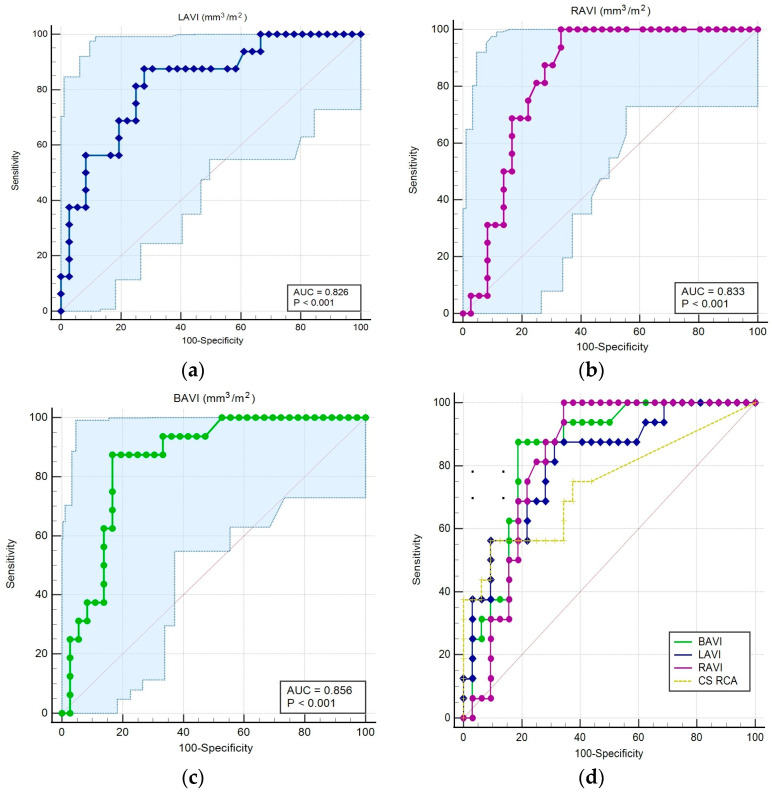
ROC analysis to evaluate the prognostic value of atrial volumes determined by CCTA. (**a**) LAVI prognostic values for AF recurrence; (**b**) RAVI prognostic values for AF recurrence; (**c**) BAVI prognostic values for AF recurrence; (**d**) ROC curve comparing atrial volumes and RCA calcium score.

ROC curve analysis revealed that indexed left, right, and bi-atrial volumes have a significant prognostic value for AF recurrence after PVI. The area under the curve (AUC) was 0.826 (95% CI: 0.69–0.91) for LAVI (*p* < 0.0001), AUC = 0.833 (95% CI: 0.70–0.92) for RAVI (*p* < 0.0001), and AUC = 0.856 (95% CI: 0.73–0.93) for BAVI (*p* < 0.0001). When analyzing the prognostic value of CS burden in the three main coronary vessels, RCA-CS had the highest prognostic value for AF. The AUC for RCA-CS was 0.733 (95% CI: 0.58–0.84, *p* = 0.004) (Figure 5). Furthermore, we conducted a comparative analysis between the ROC curves of each CCTA-derived parameter, which were similar in terms of specificity, sensitivity, and AUC values (*p* > 0.05).

**Figure 6:**
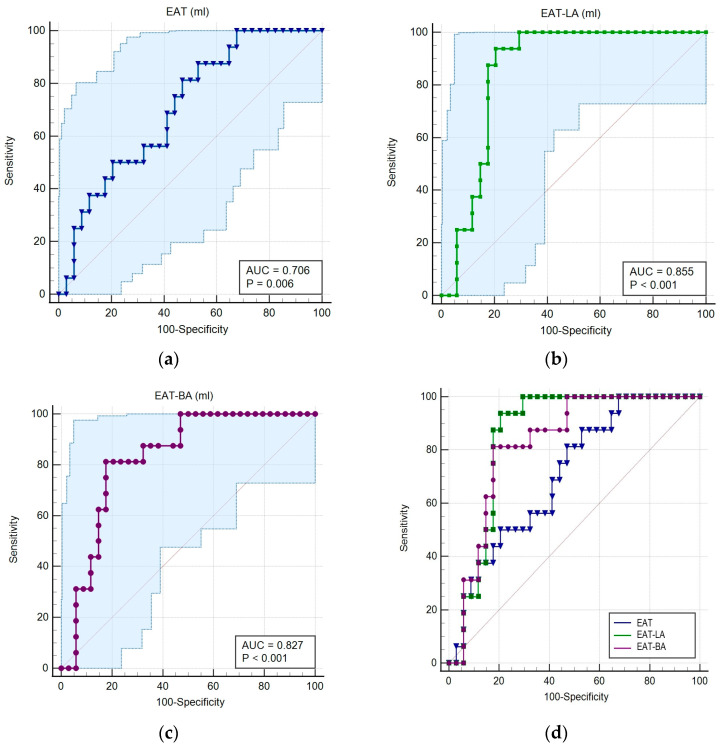
ROC analysis to evaluate the prognostic value of EAT volumes assessed by CCTA. (**a**) EAT and AF recurrence; (**b**) EAT-LA and AF recurrence; (**c**) EAT-BA and AF recurrence; (**d**) ROC curve comparison of EAT volumes.

The ROC curve analysis demonstrated that EAT has a good prognostic value for AF recurrence, with an AUC of 0.706 (95% CI: 0.56–0.82, *p* = 0.006). Analyzing the distribution of EAT in more detail, we observed that EAT located around the left atrium (EAT-LA) and bi-atrial (EAT-BA) had a significantly higher prognostic value for AF recurrence, as indicated by the ROC curves. The AUC was 0.855 (95% CI: 0.72–0.93) for EAT-AS (*p* < 0.0001) and 0.827 (95% CI: 0.69–0.91) for EAT-BA (*p* < 0.0001), both parameters demonstrating high specificity and sensitivity. Comparative analysis was performed between the ROC curves of EAT volume parameters, revealing a significant difference between EAT-LA and total EAT volume (*p* = 0.01), as well as between EAT-BA and total EAT volume (*p* = 0.02). However, there was no statistically significant difference between EAT-LA volume and EAT-BA volume (*p* = 0.3) (Figure 6).

The univariable analysis showed that 16 of the analyzed variables were in correlation with AF recurrence (Table 4). These variables include age, total coronary calcium score, RCA and LCX calcium, LA diameter, pulmonary systolic arterial pressure, and atrial indexed volumes, as well as bi-atrial indexed volumes, RCA stenosis, EAT, EAT-BA, EAT-LA. After multivariable analysis, only left, right, and bi-atrial indexed volumes, EAT-LA, and RCA stenosis assessed with cardiac CT had a positive correlation with AF recurrence after catheter ablation during 12 months of follow-up (Table 5).

## 4. Discussion

Pulmonary vein isolation by catheter ablation has an indispensable and well-accepted role in rhythm control strategies, especially in young patients with paroxysmal, symptomatic, and antiarrhythmic medication-refractory AF.

The selection of suitable patients for ablation has a main role in maintaining long-term sinus rhythm. Therefore, it is indispensable to explore factors associated with a reduced risk of arrhythmia recurrence following catheter ablation. Over the last decades, numerous studies have sought to determine clinical, laboratory, and imaging factors that can predict a positive long-term outcome. However, specific prognostic markers for lower recurrence rates have not been established [22,23].

The main objective of this study relied on investigating imaging-based parameters assessed by echocardiography, and CCTA was correlated with AF recurrence. The aim was to develop a predictive model for the recurrence of AF following PVI. 

### 4.1. General Risk Factors in Correlation with AF Recurrence

Previous research has demonstrated that age, type of AF, and the presence of mitral regurgitation occurring after PVI can independently predict AF recurrence [24]. Regarding patient demographics, cardiovascular risk factors, medical history, and type of AF, our data showed that patients with AF recurrence had a higher age compared to those who were free of AF during follow-up. There were no significant differences regarding comorbidities or AF type. A study by Sonhns et al. also found similar results. Patients with recurrence had a higher age, although not significantly (recurrence group—61 ± 5 versus no-recurrence—62 ± 10 years, *p* = 0.09). However, paroxysmal AF was more frequent in patients who did not present arrhythmia recurrence (62% versus 76%, *p* = 0.001), but the type of arrhythmia did not have significant predictive capacity during multivariable analysis [25]. 

### 4.2. Echocardiography Parameters in Correlation with AF Recurrence

In our study, the LA diameter assessed by echocardiography was one of the main indicators of AF recurrence after PV isolation, but it did not maintain its statistical significance during multivariable analysis. It is unclear whether LA dilatation is a cause or an effect of AF, as increasing LA dimensions leads to structural remodeling and hypertrophy. Several studies have reported atrial distension as a risk factor for extensive LA remodeling, which can decrease the success of the catheter ablation procedure [26,27]. In other studies, LA diameter assessed by M-mode echocardiography was a significant predictor of AF recurrence after ablation procedures [28]. 

Another echocardiographic parameter for AF recurrence is a lower left ventricular ejection fraction. In patients with heart failure, the incidence of AF increases as the LVEF decreases. The occurrence of AF is directly related to the severity of heart failure, with AF rates increasing from 10% in patients classified as New York Heart Association (NYHA) class I to 50% in those classified as NYHA class IV [29,30].

### 4.3. CCTA Parameters in Correlation with AF Recurrence

Left atrial enlargement indicates the extent of the structural remodeling at this level. CCTA evaluation of atrial anatomy and structure, as well as volumetric assessment, is a better imaging modality for patient selection and preprocedural evaluation compared to evaluating atrial dimensions using echocardiography. In addition, CCTA offers a comprehensive evaluation of coronary anatomy and atherosclerotic burden. 

On the one hand, our results showed that LA volume and the indexed LA volume are correlated with AF recurrence, which was already supported by previous studies in which both the diameter and volume were independent predictors for AF recurrence [27]. There are different smaller studies with diverse measurement protocols describing ranges for LAV and LAVI as increasing the risk of recurrence [19,31]. Using a manual measurement of the ECG-gated CCTA slices, our data demonstrated that the indexed LA volume at a cut-off value of ≥51.1 mm^3^/m^2^ has a high prognostic value for AF recurrence; the AUC for LAVI was 0.82 (*p* < 0.001). Furthermore, the multivariate analysis demonstrated that LAVI has a positive correlation with AF recurrence. These results agree with previous studies, which reported a significantly higher probability of relapse in patients with large LA volumes assessed by CCTA [25,32].

Moreover, right atrial and bi-atrial indexed volumes were significantly higher in subjects with arrhythmia recurrence, with a significant prognostic value. Patients with an increased RAVI, at a cut-off ≥35.4 mm^3^/m^2,^ were predisposed to AF recurrence (AUC = 0.83, *p* < 0.001). The results of our findings indicate that, in addition to the left atrium, the right atrium also plays a major role in AF recurrence. 

The right atrial volume was shown to be a better predictor of AF recurrence following cardioversion than the left atrial volume [33]. In addition, RA enlargement was associated with older age and persistent and permanent AF. It was independently linked to a higher risk of heart failure, cerebrovascular events, and mortality in a single-center study involving 254 subjects with non-valvular AF [34]. A study based on cardiac MRI, which included patients undergoing pulmonary vein isolation, showed that all three atrial volumes (left, right, and bi-atrial) were significantly correlated with AF recurrence after ablation, but the bi-atrial volume presented the highest AUC value for risk prediction in this context [35]. This is similar to our results, where we found significant prognostic capacity for all indexed volumes, but the bi-atrial indexed volume presented the largest area under the curve compared with LAVI or RAVI alone.

Several pathophysiological mechanisms have been postulated for the bidirectional relationship between left atrial dilatation and AF. Local inflammatory cytokines can trigger cardiac cell hypertrophy and favor the appearance of interstitial fibrosis, leading to atrial enlargement [36,37]. Uneven atrial fibrosis leads to areas of slow conduction and altered repolarization dynamics that may be the source of atrial rotors [38], which move the focus of initiation and persistence of AF from the PV to the atrium. This assumption is supported by our results. Hence, we can see that, in addition to the left atrial volume, the right atrial and bi-atrial volumes indexed by PVI have a positive correlation with AF recurrence following PVI. 

### 4.4. Role of Calcium Score and Coronary Stenosis in AF Recurrence

Several studies aimed to investigate the correlation between calcium scores and AF. Nicklas V et al. demonstrated a good correlation between a high coronary CS and an increased risk for AF in a Danish registry-based cohort study. Additionally, CS associated with other AF risk factors can aid clinicians in identifying patients with a high predisposition to AF [39]. 

However, there are few studies that have explored the role of coronary CS and non-obstructive coronary stenosis in AF or their role in predicting recurrence following PVI. We conducted a comparative analysis between the two study groups in regard to the total coronary calcium score and its distribution across the three main coronary arteries. The results demonstrated that patients with arrhythmia recurrence presented a higher total CS, which was also found in the left circumflex and right coronary arteries but not at the level of the left anterior descendant artery. There are few studies that have evaluated AF recurrence based on stenosis located at the level of the RCA. These studies have primarily focused on coronary disease with significant stenosis, which requires revascularization and results in a significant decrease in AF recurrence after ablation. In the case of patients in our studied group, stenosis located at the level of the RCA, which is responsible for vascularization of the atria in most patients, was <40%, with a cut-off value of 15% in ROC analysis. This indicates that atherosclerosis in its early stages, likely due to local inflammation, may play an important role in the occurrence of arrhythmias. Multivariable analysis performed for coronary stenosis and coronary CS demonstrated that stenosis located at the RCA also has a positive correlation with arrhythmia recurrence, unlike CS at this level.

### 4.5. The Role of EAT in AF Recurrence

The current study shows that total EAT did not present a positive correlation in multivariate analysis despite having a significant prognostic value for AF recurrence (AUC = 0.706, 95% CI: 0.56–0.82, *p* = 0.006). Similar results questioning the role of pericardial adipose tissue as an independent prognostic factor have been described in a recent study [40].

Based on the ROC analyses, EAT-AS and EAT-BA have significant prognostic value in AF recurrence, with an AUC of 0.855 (95% CI: 0.72–0.93) for EAT-LA (*p* < 0.0001) and an AUC of 0.827 (95% CI: 0.69–0.91) for EAT-BA (*p* < 0.0001). Comparing the ROC curves, it was found that EAT-AS and EAT-BA had similar prognostic values (*p* = 0.3), but there was a significant difference between EAT-LA and total EAT (*p* = 0.01), as well as between EAT-BA and total EAT (*p* = 0.02). However, in the multivariate analysis, the EAT-LA had a positive correlation with arrhythmia recurrence. From these results, it can be concluded that LAE located at the atria level has a better correlation with recurrence in comparison with total LAE. 

### 4.6. Study Limitations

Our study presented additional findings from CCTA parameters on the prognostic role of AF recurrence after PVI, but it has some limitations. Firstly, this study was conducted at a single medical center with a sample size of 80 patients, which might not have the statistical power to capture rare events or provide robust analyses for some variables. The power and sample size were not calculated in this study. By not conducting a power calculation, we did not establish the sample size based on effect sizes or significance levels, which could influence the generalizability of our findings. Consequently, it is essential to interpret our results and conduct research using larger sample sizes and power calculations to validate and expand upon our findings. Certain exclusion criteria, such as excluding patients with long-standing persistent AF, may limit the applicability of the findings to these patient groups. The second main limitation was the duration of the follow-up period and AF recurrence monitoring during this period. The 12-month follow-up period might not capture longer-term outcomes or late recurrences of atrial fibrillation. The 48-h Holter monitoring may not capture all instances of AF recurrence, as some episodes may be intermittent, and longer-term monitoring using implantable loop recorders could provide more comprehensive data.

### 4.7. Future Outlooks and Clinical Applications

With ongoing research, there is potential for the development of more accurate predictive models for the recurrence of AF following PVI using imaging-based parameters. These models could incorporate a combination of clinical, laboratory, and imaging parameters to identify patients at higher risk of AF recurrence. By refining these models, clinicians can better select suitable patients for ablation procedures, improving long-term sinus rhythm maintenance.

CCTA offers a comprehensive evaluation of atrial anatomy, structure, and volumetric assessment, along with coronary anatomy and atherosclerotic burden. As CCTA techniques improve, it may become an essential imaging modality for patient selection and preprocedural evaluation before PVI. The incorporation of CCTA findings, such as LAVI and BAVI, into predictive models can provide additional valuable information for identifying patients at a higher risk of AF recurrence. Future studies may focus on understanding the relationship between BAVI and atrial fibrosis in the context of AF recurrence. Uneven atrial fibrosis and areas of slow conduction may contribute to the initiation and persistence of AF. By considering the BAVI as a predictor, clinicians can potentially gain more insights into the overall atrial remodeling and develop targeted strategies to reduce the arrhythmic burden.

The role of CS and incipient coronary stenosis in AF recurrence is not well established; future investigations may explore this potential correlation. Understanding the association between a high CS and increased risk for AF, along with its distribution in coronary arteries supplying the atria, can provide valuable insights into the potential role of atrial ischemic episodes, local inflammatory responses, and altered electrical function in AF recurrence. Knowledge of these new findings can improve patient selection, procedural outcomes, and long-term rhythm control strategies in the management of AF. We believe that the novelty of our study is related to the study of AF recurrence in patients with documented coronary artery disease, a field that has been less investigated so far.

## 5. Conclusions

Patients with increased atrial indexed volumes, a large LA diameter, a high CS, and a high EAT before ablation present a significantly higher risk of expressing AF recurrence at 12-month follow-up. The factors with a cut-off value like LAVI of ≥51.1 mm^3^/m^2^, RAVI ≥35.4 mm^3^/m^2^, BAVI ≥87.1 mm^3^/m^2^, EAT-LA 17.3 mL, and EAT-BV 39.3 mL had good prognostic value for AF recurrence, followed by PVI. Atrial volumes and EAT-AS analyzed during the pre-procedural workup based on CCTA imaging had a positive correlation with AF recurrence. 

One of the novel findings of this study was that non-obstructive stenosis localized at the level of the RCA could be associated with a more frequent AF recurrence following catheter ablation, which will lead to further investigations. Calcium scoring at CCTA, reflecting the atherosclerotic burden at the level of coronary arteries, specifically localized on the RCA and LCX, presented higher levels in patients with AF recurrence. However, in multivariate analysis, they were not identified as independent factors for AF recurrence. Non-invasive multimodality parameters can easily be included in the pre-procedural imaging workup of patients proposed for catheter ablation. This may improve prognostic accuracy and allow better patient selection.

## Figures and Tables

**Table 1:** Baseline characteristics, cardiovascular risk factors, comorbidities, and AF type in the study population.

Variable	Group 1 AF Recurrence *n* = 23	Group 2 No AF Recurrence *n* = 57	OR (95% CI)	*p*-Value
Patient demographics				
Age (y), mean ± SD (median)	55.4 ± 9.2 (57)	60.35 ± 9.2 (59)	-	0.03
Male gender, *n* (%)	10 (43%)	38 (66%)	0.384 (0.150–1.056)	0.055
Cardiovascular risk factors				
Hypertension, *n* (%)	19 (82%)	39 (68%)	2.192 (0.709–6.584)	0.2
Diabetes, *n* (%)	3 (13%)	7 (12%)	1.071 (0.279–4.646)	0.9
Obesity, *n* (%)	9 (39%)	18 (31%)	1.393 (0.517–3.941)	0.7
Smoking, *n* (%)	3 (13%)	5 (8%)	1.56 (0.382–6.854)	0.6
Dyslipidaemia, *n* (%)	3 (13%)	13 (23%)		0.3
Medical history				
Coronary artery disease, *n* (%)	3 (13%)	4 (7%)	1.988 (0.463–7.873)	0.4
Prior Stroke, *n* (%)	0 (0%)	2 (3%)	0 (0–5.377)	0.9
Pulmonary disease, *n* (%)	3 (13%)	2 (3.5%)	4.125 (0.7794–24.05)	0.1
Type of AF				
Paroxysmal, *n* (%)	8 (35%)	29 (51%)	0.514 (0.1822–1.392)	0.2
Persistent, *n* (%)	15 (65%)	28 (49%)		
Current medication				
Lipid-lowering medical treatment, *n* (%)	12 (52%)	41 (71%)	0.4257 (0.168–1.136)	0.09
Antihypertensive medical treatment, *n* (%)	15 (65%)	33 (58%)	1.364 (0.499–3.875)	0.7
Antiarrhythmic medical treatment, *n* (%)	15 (65%)	43 (77%)	0.566 (0.208–1.755)	0.4
Anticoagulant medical treatment, *n* (%)	17 (73%)	45 (79%)	0.755 (0.253–2.475)	0.8

**Table 2:** Echocardiographic parameters and AF recurrence followed PVI.

Variable (Mean ± SD)	Group 1 AF Recurrence *n* = 23	Group 2 No AF Recurrence *n* = 57	*p*-Value
LVEDD (mm)	50.35 ± 10.71	50.77 ± 4.01	0.2
LVESD (mm)	34.09 ± 5.99	33.12 ± 5.17	0.4
LVS thickness (mm)	11.96 ± 1.49	11.46 ± 1.31	0.1
PW thickness (mm)	11.22 ± 1.83	10.86 ± 1.31	0.3
LVEF (%)	51.26 ± 4.58	53.14 ± 5.54	0.03
RV diameter (mm)	36.13 ± 5.39	35.41 ± 4.41	0.9
LA diameter (mm)	45.3 ± 4.29	41.25 ± 5.94	0.003
PAPs (mmHg)	28.28 ± 7.7	24.53 ± 6.49	0.01
E (cm/s)	66.87 ± 18.65	74.61 ± 19	0.1
A (cm/s)	71.91 ± 17.17	67.32 ± 18.47	0.3
DT (ms)	199.1 ± 31.57	195.9 ± 39.49	0.7

VEDD—left ventricular end-diastolic dimension; LVESD—left ventricular end-systolic dimension; LVS—left ventricular septum; PW—posterior wall; LVEF—left ventricular ejection fraction; RV—right ventricle; LA—left atrium; E—mitral early diastolic velocity; A—mitral late diastolic velocity; DT—deceleration time.

**Table 3:** CCTA features are associated with AF recurrence after ablation.

Variable, Mean ± SD, (Median)	Group 1 AF Recurrence *n* = 23	Group 2 No AF Recurrence *n* = 57	*p*-Value
LAV (mm^3^)	108.7 ± 21.25 (106.3)	83.79 ± 19.94 (80.1)	0.0002
LAVI (mm^3^/m^2^)	61.38 ± 11.12 (62.15)	46.34 ± 12.27 (45.45)	0.0001
RAV (mm^3^)	85.18 ± 15.86 (79.2)	67.25 ± 19.2 (64.25)	0.0002
RAVI (mm^3^/m^2^)	43.07 ± 5.64 (41.55)	33.66 ± 9.97 (30.9)	0.0001
BAVI (mm^3^/m^2^)	98.23 ± 14.44 (96.65)	76.48 ± 17.61 (72.3)	0.0001
CS total (UI)	136.8 ± 37.29 (69.35)	31.56 ± 7.75 (7.8)	0.02
CS LAD (UI)	44.86 ± 15 (3.7)	18.5 ± 5.47 (25.4)	0.09
CS LCX (UI)	20.97 ± 7.71 (0.65)	2.18 ± 0.9 (0)	0.03
CS RCA (UI)	73.97 ± 22.83 (46.35)	8.88 ± 3.08 (0)	0.005
LAD stenosis (%)	20.03 ± 3.66	12.94 ± 2.16	0.09
LCX stenosis (%)	12.91 ± 4.34	5.17 ± 1.69	0.07
RCA stenosis (%)	25.38 ± 4.1	9.76 ± 2.32	0.001
EAT (mL)	202.8 ± 56.3	156.0 ± 66.76	0.01
EAT-LA (mL)	25.55 ± 6.37	15.54 ± 8.44	<0.0001
EAT BA (mL)	52.24 ± 12.54	33.84 ± 15.9	0.0001

AV—left atrial volume; RAV—right atrial volume; LAVI—left atrial volume index; RAVI—right atrial volume index; BAVI—bi-atrial volume index; CS—calcium score; LAD—anterior descendent artery; LCX—circumflex artery; RCA—right coronary artery; EAT—epicardial adipose tissue; EAT-LA—left atrial epicardial adipose tissue; EAT-BA—bi-atrial epicardial adipose tissue.

**Table 4:** ROC curve analysis for CCTA imaging markers in correlation with AF recurrence.

Study Population (Atrial Volumes and Calcium Score)								
Parameter	AUC	95% CI for AUC	*z*-Statistic	Youden Index	Cut-Off Value for AF Recurrence	Sensitivity %	Specificity %	*p*-Value
LAVI (mm^3^/m^2^)	0.826	0.69–0.91	5.23	0.597	>51.1	87.50	72.22	<0.0001
RAVI mm^3^/m^2^)	0.833	0.70–0.92	5.99	0.666	>35.4	100.00	66.67	<0.0001
BAVI (mm^3^/m^2^)	0.856	0.73–0.93	6.72	0.708	>87.1	87.50	83.33	<0.0001
CS LAD (UI)	0.648	0.49–0.77	1.65	0.34	>31.2	50.00	84.85	0.09
CS LCX (UI)	0.634	0.48–0.76	1.63	0.28	>1.1	50.00	78.79	0.1
CS RCA (UI)	0.733	0.58–0.84	2.82	0.47	>28.8	56.25	90.91	0.004
Total CS	0.699	0.55–0.82	2.16	0.43	>135.2	43.75	100	0.03
LAD stenosis (%)	0.683	0.53–0.80	2.11	0.34	>25	46.67	88.24	0.035
LCX stenosis (%)	0.656	0.50–0.79	1.68	0.33	>10	54.55	79.41	0.092
RCA stenosis (%)	0.759	0.65–0.89	4.18	0.47	>13	80.00	67.65	<0.0001
EAT (mL)	0.706	0.56–0.82	2.72	0.34	>144.1	87.50	47.06	0.006
EAT-LA (mL)	0.855	0.72–0.93	6.47	0.73	>17.3	93.75	79.41	<0.0001
EAT-BA (mL)	0.827	0.69–0.91	5.53	0.63	>39.3	81.21	82.35	<0.0001

**Table 5:** Uni- and multivariable logistic analysis for predictors of AF recurrence during the 1-year follow-up.

Univariable Analysis			
Variable	OR (95% CI)	95% CI for OR	*p*-Value
Age	1.06	1.0–1.13	0.03
Male gender	0.38	0.13–1.02	0.058
Paroxysmal AF	1.9	0.72–5.49	0.1
Total CS	1.01	1.00–1.02	0.007
CS LAD	1.01	0.99–1.03	0.07
CS LCX	1.04	1.01–1.09	0.02
CS RCA	1.03	1.01–1.05	0.01
Diabetes	1.07	0.21–4.28	0.9
Hypertension	1.30	0.76–2.27	0.3
Obesity	1.88	0.80–4.44	0.1
Smoking	1.56	0.29–6.97	0.5
Coronary artery disease	1.25	0.05–13.70	0.8
Pulmonary disease	4.12	0.63–33.08	0.1
LVEF	0.93	0.84–1.02	0.1
LVEDD	0.99	0.91–1.07	0.7
LVESD	1.03	0.94–1.13	0.4
LA diameter	1.15	1.04–1.29	0.006
RV diameter	1.03	0.93–1.14	0.5
LV diastolic dysfunction	2.66	0.99–7.43	0.053
PAPs	1.07	1.0–1.16	0.04
LAV	1.05	1.02–1.10	0.001
RAV	1.05	1.01–1.10	0.008
LAVI	1.12	1.05–1.2	0.001
RAVI	1.12	1.04–1.23	0.004
BAVI	1.07	1.03–1.13	0.0009
EAT	1.01	1.0–1.02	0.02
EAT-LA	1.15	1.06.1.27	0.001
EAT-BA	1.07	1.03–1.13	0.002
LAD stenisis	1.05	1.00–1.1	0.05
LCX stenosis	1.05	0.9–1.12	0.06
RCA stenosis	1.07	1.03–1.1	0.001
Multivariable regression analysis			
**Variable**	**OR**	**95% CI for OR**	***p*-Value**
Age	0.97	0.76–1.21	0.7
PAPs	1.00	0.82–1.15	0.9
Total CS	0.71	0.23–1.18	0.5
CS LCX	1.39	0.89–4.46	0.4
CS RCA	1.46	0.89–4.59	0.4
RCA stenosis	1.18	1.04–1.51	0.04
LAVI	1.21	1.06–1.52	0.02
RAVI	1.12	1.01–1.28	0.04
BAVI	1.09	1.03–1.20	0.01
EAT	0.98	0.95	0.1
EAT-LA	1.24	1.07–1.49	0.007

Variables correlated with others were not included in the multivariable regression analysis (LA, RA volumes, and EAT-BA were excluded).

## Data Availability

The data presented in this study are available on request from the corresponding authors. The data are not publicly available due to privacy and ethical restrictions (containing information that could compromise the privacy of the study subjects).
